# Novel Role of the Epstein-Barr Virus Encoded Deubiquitinating Enzyme (BPLF1) in mTOR-Mediated Cell Growth and Proliferation Pathways

**DOI:** 10.3390/v17081139

**Published:** 2025-08-20

**Authors:** Rachel Mund, Sage L. Atkins, Anwen Cao, Aminatou Diallo, Christopher B. Whitehurst

**Affiliations:** 1 Department of Pathology, Microbiology and Immunology, New York Medical College, New York, NY 10595, USA; rmund@student.touro.edu (R.M.); anwenc06@gmail.com (A.C.); aminatoud14@gmail.com (A.D.); 2 Department of Microbiology and Immunology, University of North Carolina Chapel Hill, Chapel Hill, NC 27599, USA; sage.atkins@emory.edu

**Keywords:** Epstein-Barr Virus, BPLF1, deubiquitinating enzyme, mTOR, mTORC1, DUB, EBV

## Abstract

Epstein-Barr Virus (EBV) is a causative agent of infectious mononucleosis and is strongly associated with Burkitt lymphoma, Hodgkin lymphoma, and nasopharyngeal carcinoma. EBV encodes a deubiquitinating enzyme, BPLF1, which is important for infectious virus production, B-cell immortalization, and tumorigenesis. To elucidate BPLF1’s role, an affinity-based mass spectrometry screen was performed, which suggested that BPLF1 and mTOR interact. mTOR, a critical mediator within cellular signaling cascades and oncogenesis, exists in two distinct complexes: mTOR Complex 1 (mTORC1) and mTOR Complex 2 (mTORC2). Here, we show that BPLF1 has direct deubiquitinating (DUB) activity on mTOR, removing both K48- and K63-ubiquitin linkages. Additionally, WT BPLF1 decreased mTORC1 localization to the lysosome and decreased the phosphorylation of mTORC1 downstream effectors, 4E-BP1 and S6K1. BPLF1 also had DUB activity on Raptor and Rictor, which have both been shown to preferentially cause the formation of mTORC2 over mTORC1 when not ubiquitinated. Immunoprecipitation of mTOR shows decreased mTORC1 formation in the presence of WT BPLF1. Importantly, treatment with rapamycin, an mTORC1 inhibitor, increased infectious virus production, while JR-AB2-011, an mTORC2 inhibitor, reduced infectious virus production. Taken together, these data demonstrate that BPLF1’s effect on the mTOR signaling cascade regulates cellular and viral processes during EBV infectivity and replication.

## 1. Introduction

EBV was first discovered in 1964 by Dr. Anthony Epstein and Yvonne Barr and was the first human tumor virus identified [1]. EBV infects ~90% of the global population. It is an etiological agent of infectious mononucleosis and a pathogenic driver of several cancers, including Burkitt’s lymphoma, Hodgkin’s lymphoma, and nasopharyngeal carcinoma (NPC) [2,3,4]. Additionally, EBV can cause fatal immunoblastic lymphomas in individuals with immune deficiencies and has been linked to multiple sclerosis, although the underlying mechanisms remain unclear [5,6].

One central tool that EBV uses to manipulate host mechanisms are post-translational modifications (PTMs). PTMs allow the virus to modify endogenous protein function and aid it in its evasion of host detection mechanisms. One PTM that many viruses, including EBV, utilize is the ubiquitin-controlled substrate regulation and degradation pathway [7,8].

Protein substrates can undergo either monoubiquitination or polyubiquitination. Ubiquitin has seven lysine residues (K6, K11, K27, K29, K33, K48, and K63) that serve as attachment points for polyubiquitin chain formation [9]. Among these, Lysine-48 (K48) and Lysine-63 (K63) have been the most extensively studied and have distinct signaling functions. K48-linked ubiquitination typically marks proteins for proteasomal degradation, whereas K63-linked ubiquitination is primarily associated with regulatory roles in cellular processes [9,10].

Endogenously, ubiquitin is removed from substrates by more than 100 cellular deubiquitinating enzymes (DUBs), which play crucial roles in regulating ubiquitin-mediated pathways [11]. In 2005, researchers identified a conserved deubiquitinating enzyme (DUB) encoded by the Herpesviridae family [12,13]. BPLF1, the DUB of EBV, is a late lytic cycle gene and tegument protein, with its deubiquitinating activity localized in the N-terminal region. It can cleave both K48- and K63-linked polyubiquitin chains and remove ubiquitin from monoubiquitinated substrates. Notably, mutation of the active site cysteine abolishes its DUB activity [14,15].

BPLF1 has been shown to be important for a variety of EBV functions. BPLF1 is a potential oncogenic factor due to its ability to interfere with DNA repair and interact with various cellular DNA repair proteins [14,15,16]. BPLF1 also contributes to the ability of EBV to evade immune detection [17,18,19,20].

Furthermore, BPLF1 plays a critical role in infectious virus production, B-cell immortalization, and tumor development [14,21]. Studies have shown that a BPLF1 knockout virus produces 90% fewer infectious particles than the wild-type virus [14,21,22]. Moreover, EBV’s hallmark ability to transform B-cells in culture is both reduced and delayed in the absence of BPLF1 [14,21]. Furthermore, humanized mice infected with the BPLF1 knockout virus exhibited longer survival rates and a lower incidence of splenic tumors [21].

To further elucidate BPLF1’s role in EBV-associated processes, we performed an affinity-based mass spectrometry screen, which suggested an interaction between BPLF1 and the mechanistic target of rapamycin (mTOR). mTOR, a highly conserved protein kinase and critical mediator within cellular signaling cascades, is present in the cell in complexes called mTOR Complex 1 (mTORC1) and mTOR Complex 2 (mTORC2). mTOR regulates cell growth and metabolic processes, and dysregulation of its pathway is implicated in oncogenic processes [23].

**Figure 1:**
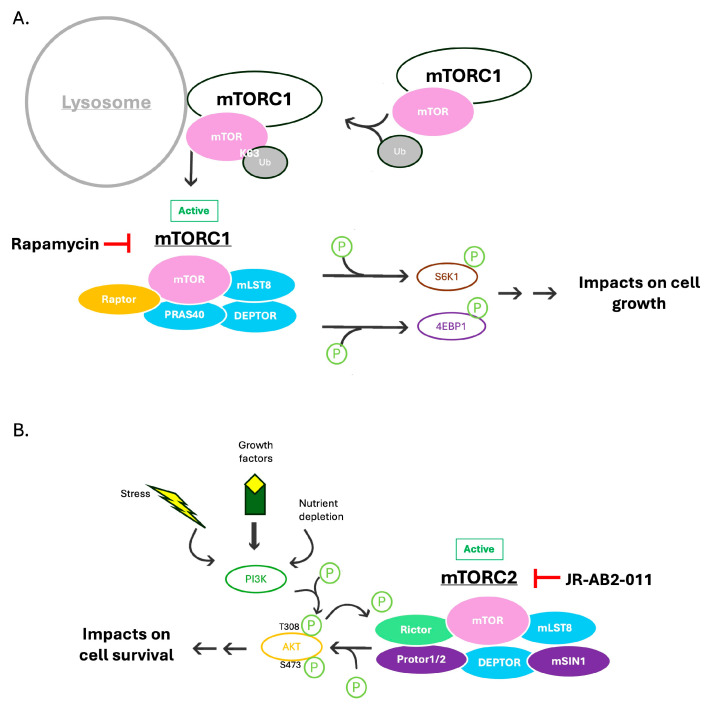
Overview of mTOR complex activation. (**A**). mTORC1 activation. mTORC1 is activated at the lysosome when mTOR is K63-ubiquitinated. Once activated, mTORC1 acts on downstream effectors, phosphorylating S6K1 and 4E-BP1. This impacts cell growth. mTORC1 is specifically inhibited by rapamycin. (**B**). mTORC2 activation. mTORC2 primarily acts on AKT through phosphorylation at its S473 site. mTORC2 activation impacts cell survival. mTORC2 is specifically inhibited by JR-AB2-011.

mTORC1 has three key proteins: mTOR, Raptor (regulatory-associated protein of mTOR), and mLST8 (mammalian lethal with Sec13 protein 8, GβL) (Figure 1A) [24,25,26]. Raptor promotes complex localization to the lysosome by binding to the TOR signaling (TOS) motif found in many mTORC1 substrates [27]. mLST8 contributes to the catalytic domain of mTORC1, although it is not essential for mTORC1’s critical functions [28]. mTORC1 also has two protein subunits that play inhibitory functions: PRAS40 (proline-rich Akt substrate of 40 kDa) and DEPTOR (DEP domain-containing mTOR-interacting protein) (Figure 1A) [29,30,31].

mTORC1’s catalytic function is typically characterized by the phosphorylation of its downstream effectors, p70S6 Kinase 1 (S6K1), and eIF4E Binding Protein (4E-BP). S6K1 is phosphorylated at Thr389 (hydrophobic motif), which allows PDK1 to activate it. Once activated, S6K1 promotes mRNA translation [32]. 4E-BP is phosphorylated by mTORC1, which causes it to release its inhibition of eIF4E, allowing 5′ cap-dependent translation [33]. mTORC1 is also involved in the regulation of autophagy and appears to play an important role in regulating the ubiquitin proteasome system. It has been shown that mTORC1 is inhibited under starvation conditions, which leads to increased proteasomal targeting via the ubiquitination of substrates [34].

mTORC1 activation depends on high-nutrient conditions, leading to the promotion of energy storage in tissues such as the liver and muscle [29]. mTORC1 is also sensitive to intracellular and environmental stresses, such as low ATP levels, hypoxia, or DNA damage, which allow AMPK to inhibit mTORC1 [35]. mTORC1 is activated at the lysosome, where it undergoes a conformational change to its active state. Of note, mTORC1 only localizes to the lysosome when mTOR is K63-ubiquitinated by the E3 ligase TRAF6 (Figure 1A) [36].

mTORC2, like mTORC1, contains mTOR, mLST8, and DEPTOR. However, instead of Raptor, mTORC2 includes Rictor (rapamycin-insensitive companion of mTOR) (Figure 1B) [37]. mTORC2 also has regulatory subunits mSin1 and Protor1/2 [38,39]. mTORC2 activation depends on insulin/PI3K signaling. Once activated, mTORC2 has phosphorylation activity on the AGC kinase family (PKA/PKG/PKC) as well as on Akt, which it phosphorylates and activates, resulting in downstream effects on cell proliferation and survival (Figure 1B) [40,41].

There is robust evidence that EBV pathogenesis involves the manipulation of the mTOR pathway. Several EBV latency proteins have been identified as interacting with components of the mTOR pathway, including LMP1’s effect on the phosphorylated forms of mTOR, P70S6K, and 4E-BP1, as well as on Glut-1 transcription via the mTORC1/NF-κB signaling pathway to promote growth in NPC tumors [42,43]. Furthermore, LMP1 was found to activate both mTORC1 and mTORC2 pathways in EBV-driven NPC, and the knockdown of mTORC1 increased NPC treatment susceptibility in previously treatment-resistant NPC [44]. It was further shown that NPC tumor initiation is dependent on mTORC2 signaling, as the knockdown of Rictor or mTOR prevented NPC tumor formation [44]. Additionally, LMP2A has been shown to activate PI3K/Akt, leading to mTOR-mediated downstream inhibition of 4E-BP1 and upregulation of c-Myc, and plays a role in the formation of EBV-induced vasculogenic mimicry (tumor that mimics blood vessels) [45,46].

mTORC1 and mTORC2 inhibitors have been used to treat various cancers with varying efficacy. While mTORC1 inhibitors have been shown to have limited therapeutic efficacy in a number of cancers, it is thought that inhibiting mTORC2 could inhibit angiogenesis and promote the recruitment of immune cells to tumors, allowing for greater efficacy with inhibition [47]. Treatment with rapamycin (mTORC1 inhibitor) affects lytic replication in a cell type-dependent manner, decreasing lytic replication in B-cell lines but increasing lytic replication in epithelial cell lines [48]. Further, Manassantin B (an mTORC2 inhibitor) suppresses EBV lytic replication by disrupting the mTORC2–PKC/AKT signaling pathway, ultimately inhibiting EBV immediate-early gene BZLF1 expression [49]. Selectively inhibiting PI3Kδ and downstream inhibition of Akt activation in EBV+ B cell lymphomas enhanced the anti-proliferative effects of rapamycin on these lymphomas [50]. The feedback that occurs between mTORC1 and mTORC2 and the pathway’s importance to cell survival make the study exceedingly complex [47].

In this study, BPLF1’s important yet complex role on the mTOR signaling cascade is explored. Here, we found that BPLF1 deubiquitinates mTOR, resulting in the disruption of mTORC1 complex formation, localization, and downstream signaling. Additionally, studies have demonstrated that inhibition of mTORC1 promotes infectious virus production. BPLF1’s ability to regulate both cellular and viral processes may be through its manipulation of the mTOR pathway during EBV infection and replication.

## 2. Materials and Methods

### 2.1. Cell Lines, Growth, and Transfection

HEK 293T, 293EBV+ (green fluorescent protein [GFP]-tagged recombinant B95.8 EBV) [51], and HeLa cells were cultured in Dulbecco’s modified Eagle medium (Corning, Tewksbury, MA, USA) supplemented with 10% fetal bovine serum, Antibiotic-Antimycotic (100X) (Sigma Aldrich, Saint Louis, MO, USA). Raji cells were cultured in RPMI supplemented with 10% fetal bovine serum, Antibiotic-Antimycotic (100X) (Sigma Aldrich). Transfections were performed using the Effectene transfection reagent (Qiagen, Hilden, Germany) and followed the protocol provided by the manufacturer. Transfected cells were harvested after 24 h for immunofluorescence and 48 h for all other experiments. Plasmids used: FLAG-tagged BPLF1 constructs [14], BZLF1, and GP110 [52]. Purchased from Addgene: pcDNA3-AU1-mTOR-E2419K (Plasmid #19994), HA-Ubiquitin (Plasmid #18712), pRK5-HA-Ubiquitin-K48 (Plasmid #17605), pRK5-HA-Ubiquitin-K63 (Plasmid #17606), pcDNA3-Flag mTOR wt (Plasmid #26603), HA Raptor (Plasmid #8513), myc-Raptor (Plasmid #1859), myc-Rictor corrected (Plasmid #11367).

### 2.2. BPLF1 Full Length (FL) and BPLF1 FL C61S Construction

DNA was extracted from 293EBV+ cells (B95-8) and used as a template [51]. The full-length DNA sequence coding for BLF1 was amplified by PCR using PrimeStar GLX polymerase (Takara, Shiga, Japan) and the following primers: BPF1 FWD 5′ GTTTAAACTTAAGCTTATGAGTAACGGCGACTGG 3′, which contains a HindIII restriction enzyme site. BPLF1 REV 5′ AAACGGGCCCTCTAGATTACTTATCGTCGTCATCCTTGTAATCCAGATACAAAAACTTGAGTCTC 3′, which introduces an XBa1 site and FLAG tag DNA sequence.

Touchdown PCR was performed as follows: 98 °C for 3 min, followed by 15 cycles of 98 °C for 10 s, 75–60 °C for 10 s (decreasing one degree/cycle), and 68 °C for 10 min, followed by 25 cycles of 98 °C for 10 s, 60 °C for 10 s, and 68 °C for 10 min and a final 10 min extension at 68 °C. The PCR products were gel-purified, extracted, cut with XbaI and HindIII, and ligated into pcDNA3.1. The same restriction sites were used to insert into pFLAG CMV-2 (N-terminal FLAG) and transformed into DH10B cells. The entire gene was sequenced and verified. Expression was observed at the correct molecular weight after transfection into 293T cells and probing with the FLAG antibody.

A full-length BPLF1 DNA construct with the C61S mutation was also created. Previously, a C61S inactive mutant of BPLF1 containing the first 246 amino acids was constructed [14]. A fragment containing the C61S mutation was removed with BglII and HindIII from BPLF1 C61S and placed into the same sites of the full-length BPLF1 to generate full-length BPLF1 C61S. Expression was verified by immunoblot.

Enzymatic activity of both the BPLF1 FL and BPLF1 FL 1-246 construct was confirmed using an in vitro ubiquitin rhodamine 110 assay. Ubiquitin Rhodamine 110 (South Bay Bio) was incubated with affinity purified full length BPLF1 (Supplemental Figure S1) affinity purified full length BPLF1 C61S or for 10 min in reaction buffer (50 mM Hepes pH 7.4, 0.5 mM EDTA, 100 mM NaCl, 1 mM DTT, 0.1 mg/mL BSA, and 0.01% Tween 20) and fluorescence was measured on a Gemini XPS plate reader (excitation at 485 nm, emission at 535 nm).

### 2.3. Construction of Untagged BPLF1 1-246 and BPLF1 1-246 C61S Plasmids

Previously, a shortened plasmid of BPLF1 with only the first 246 amino acids and a C61S inactive mutant of BPLF1 containing the first 246 amino acids were constructed [14]. A fragment containing the BPLF1 shortened protein or C61S mutated short protein, without the FLAG tag, was removed with BglII and HindIII from the shortened plasmid and placed into the same sites of the empty PCDNA3 vector plasmid to create an untagged BPLF1 protein. Expression was verified by immunoblotting and sequencing.

### 2.4. Mass Spectrometry Screen

BPLF1 1-246 and BPLF1 FL were transfected into 293EBV+ cells and harvested after 30 h. Affinity immunoprecipitation was performed using FLAG M2 antibody (Sigma). Samples were resuspended in 50 mM Tris, pH 7.5, and 150 mM NaCl, and LC-MS/MS was performed by the Michael Hooker Metabolomics and Proteomics Core at the University of North Carolina at Chapel Hill to identify interacting partners.

The detected hits in the mass spectrometry were quantified and calculated using Log2 of the fold change of the sample with BPLF1 overexpression vs. the control, which had no BPLF1 expressed. Data were processed using MaxQuant and searched against the Uniprot human and EBV databases. Label-free quantitation (LFQ) was used for relative quantitation. Using the Log2 LFQ intensities from each sample, log2(fold change, FC) was calculated for −/+ virus FLAG-BPLF1 vs. control (no BPLF1)and −/+ FLAG-BPLF1_1-246 vs. control (no BPLF1). Log2(FC) > 1 was considered significant.

### 2.5. In-Vitro DUB Assay

Cells transfected with HA-Ub and AU1-mTOR were harvested, and an IP for AU1 was performed. Western blotting was performed as described above to detect Ub of mTOR. The IP products for the sample with overexpressed mTOR and Ub were split into two, and half was incubated with 1 μM purified N-terminal BPLF1 or just reaction buffer (50 mM Tris, pH 7.5, 2 mM ATP, and 2 mM dithiothreitol (DTT)) at 37 °C for 10 min. Western blot analysis was performed to assess whether the purified BPLF1 protein removed Ub from mTOR.

### 2.6. Western Blots

Cell lysates with 6X denaturing sample buffer were boiled for 10 min to denature the proteins. The samples were then run on a 4–15% SDS-PAGE premade Bio-Rad gel and transferred to Immobilon transfer membrane (Millipore, Darmstadt, Germany). The membranes were blocked with 5% milk in Tris-buffered saline-Tween 20 and incubated with the appropriate primary antibody overnight at 4 °C. The nitrocellulose membrane was then washed and probed with either goat or rabbit anti-mouse antibody at 1:3000 in 5% milk for 1 h at room temperature. Bands were visualized using an enhanced chemiluminescence reagent (ThermoScientific, Waltham, MA, USA) using an iBright 1500 instrument (Invitrogen, Waltham, MA, USA). Average band intensities from biological replicates were determined using ImageJ (version 1.54m).

Primary Antibodies used: From cell signaling mTOR (1:1000), Rictor (1:1000), Raptor (1:1000), p70S6K1 (1:1000), p-p70S6K (1:1000), 4E-BP1 (1:1000), p-4E-BP1 (1:1000), AKT (1:1000), p-AKT (1:1000). Myc (1:200) and HA (1:200) were purchased from Santa Cruz Biotechnology. From Sigma Aldrich FLAG (1:3000), AU1 (1:1000). Custom made for lab BPLF1 (1:200).

Secondary Antibodies used: Rabbit HRP, Mouse HRP, fluorescent anti-rabbit (650 nm), fluorescent anti-mouse (488 nm).

### 2.7. Immunoprecipitations

Transfected cells were harvested after 48 h, rinsed once with ice-cold PBS, and lysed in ice-cold lysis buffer (mTORC1 IPs: 40 mM HEPES [pH 7.4], 120 mM NaCl, 1 mM EDTA, 10 mM pyrophosphate, 0.3% CHAPS, and one tablet of EDTA-free protease inhibitors [Roche] per 50 mL; all other IPs: 30 mM NaCl, 1% NP-40, 10 mM Tris, pH 7.5, 1× protease inhibitor cocktail tablet [Roche], 5 mM DTT, and 1 mM phenylmethylsulfonyl fluoride).

Cells were lysed by freeze-thawing (three times), and the soluble fractions of the cell lysates were isolated by centrifugation at 8600× *g* for 10 min. A 50% slurry of magnetic protein G-sepharose (20 μL) was added to each sample for preclearing. Primary antibodies were added to the lysates and incubated at 4 °C for 1 h with rotation. Next, 30 μL of a 50% slurry of magnetic protein G-sepharose was added, and incubation was continued overnight. The immunoprecipitates were washed three times with lysis buffer. Cell extracts or immunoprecipitated proteins were denatured by adding 100 μL of sample buffer and boiling for 10 min. Western blotting was performed as previously described.

### 2.8. Infection and Flow Cytometry

Infectious virus was created by inducing lytic reactivation of 293EBV+. This was accomplished by transfecting both BZLF1 and gp110 into the cells using Effectene, as described above. Drug conditions were added as described in each experiment, and the media was changed, and new drugs were added at 24 h. Supernatant fluids were harvested 48 h post-transfection and cleared of cellular debris by centrifugation at 500× *g* for 5 min. Virus was concentrated to equivalent volumes using an Amicon Ultra 15 10-kDa-molecular-mass-cutoff filter (Millipore) by centrifugation at 2500× *g*. For viral titration, 100 µL of cleared and concentrated supernatant fluids was placed on Raji cells and treated with 50 ng/mL phorbol-12-myristate-3-acetate and 3 mM sodium butyrate 24 h after infection. At 48 h, infectious titers were determined by detecting GFP-encoding EBV genomes using flow cytometry. Drugs. Rapamycin was purchased from Thermo Scientific, and JR-AB2-011 was purchased from MedChem Express. Infectious virus production in the presence of Rapamycin and JR-AB2-011 was performed in biological duplicates and assessed in technical duplicates using flow cytometry.

### 2.9. Real-Time PCR

The supernatant fluid (100 µL) was treated with Dase for 1 h at 37 °C and then neutralized with EDTA. Viral genomic DNA was extracted using a DNeasy kit (Qiagen). The extracted DNA was analyzed using real-time PCR with a Quant 5 (Applied Biosystems, Foster City, CA, USA) real-time PCR system. Primer and probe sequences were EBVW-1 (5′-GCAGCCGCCCAGTCTCT-3′), EBVW-2 (5′-ACAGACAGTGCACAGGAGCCT-3′), and EBVW-FAM (5′-6-carboxyfluorescein-AAAAGCTGGCGCCCTTGCCTG-6-carboxytetramethylrhodamine-3′). The PCR conditions were as follows: 50 °C for 2 min, 95 °C for 10 min, 40 cycles at 95 °C for 15 s, and 60 °C for 1 min.

### 2.10. Lysosomal Fractionation

Lysosomal fractions were isolated using the Invent Biotechnology Lysosome Isolation Kit, following the manufacturer’s instructions. Briefly, transfected cells were harvested, rinsed with cold PBS, resuspended in the provided lysis buffer, and homogenized by passing through a filter. After differential centrifugation, the lysosomal fraction was collected and further purified according to the manufacturer’s protocol. The purity of the lysosomal fraction was assessed by western blotting using antibodies against lysosomal marker (LAMP2), as well as markers for cytosolic components (B-actin).

## 3. Results

### 3.1. BPLF1 Interactome

**Figure 2:**
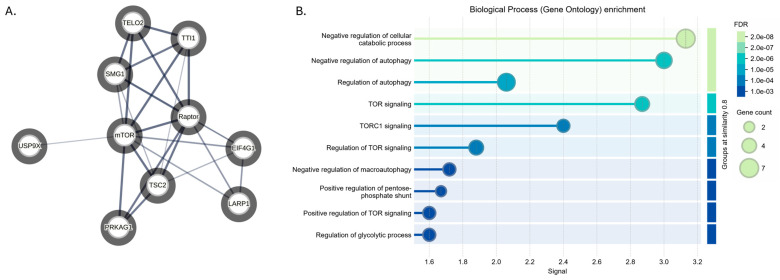
mTOR signaling pathway-related proteins that interact with BPLF1. (**A**). STRING analysis revealed several BPLF1-interacting partners known to be associated with mTOR signaling: mTOR, Raptor, TelO2, TTI1, SMG1, TSC2, EIF4G1, PRKAG1, LARP1, and USP9X. The thickness of the line denotes the strength of the known data for the interactions between the nodes. The shaded circle indicates an interaction with the prey protein BPLF1. (**B**). STRING-generated gene ontology enrichment for BPLF1-interacting partners in the mTOR pathway and related signaling.

Previous studies have demonstrated the importance of BPLF1 in infectivity, viral replication, immortalization, DNA repair, immune evasion, and tumor formation [14,21,22]. Therefore, given the vital role of BPLF1 in viral function, affinity-based mass spectrometry analysis of BPLF1 was conducted to identify potential interacting partners. To this end, Flag-tagged BPLF1 1-246 [14] (shortened BPLF1 construct with the catalytically active region) and a full-length BPLF1 construct (BPLF1 FL) were overexpressed in 293EBV+ cells [51] with and without the induction of the viral lytic cycle (Supplementary Figure S1). Immunoprecipitations were performed using anti-FLAG antibody and analyzed via LC-MS/MS. The mass spectrometry screen revealed hundreds of potential interacting partners involved in various pathways of cell processes (Supplementary Table S1). It is important to note that interacting partners identified through mass spectrometry may be either direct or indirect. Indirect interactions involving secondary proteins or protein complexes can also be detected. STRING analysis was performed, and several significant interacting partners of the mTOR pathway were identified, as indicated in Figure 2A. Figure 2B shows the breakdown of mTOR-related pathways involving the BPLF1 interacting proteins, providing insight into where BPLF1 might be having effects.

The identified interacting partners of BPLF1 that were implicated in the mTOR complex and regulation pathways are listed in Table 1. mTOR was an interacting partner found to interact with both BPLF1 1-246 and BPLF1 FL with and without the induction of the viral lytic cycle. As discussed in the introduction, mTOR is a kinase responsible for controlling cellular metabolism, catabolism, immune responses, autophagy, survival, proliferation, and migration to maintain cellular homeostasis, which is vital to the mTOR signaling pathway. Raptor (regulatory-associated protein of mTOR) is a protein that, as its name implies, is responsible for the regulation and control of mTORC1 formation and signaling. Interestingly, interactions between Raptor and BPLF1 were only found in samples without viral induction (Table 1, columns 1 and 2 vs. 3 and 4). Tel2 (Telomere Maintenance 2) and TTI1 (Telo2-interacting protein 1), which function in regulating telomeres and the DNA damage response, also help stabilize mTOR complexes and regulate mTOR signaling [53,54]. SMG1 (serine/threonine-protein kinase SMG1) is an important protein in mRNA decay, DNA damage response, and telomere maintenance, and has been shown to have a function related to the mTOR signaling pathway [55].

Further, several additional proteins involved in the mTOR signaling pathway were found to interact with BPLF1, downstream or indirectly, including TSC2, EIF4G1, PRKAG1, LARP1, and USP9X (Table 1). TSC2 is a negative regulator of mTORC1, as it downregulates the activation of Rheb, a small GTPase that directly activates mTORC1 [56]. EIF4G1 is a downstream effector of the mTORC1 pathway. Activated mTORC1 phosphorylates and inactivates 4E-BP1, a repressor that binds to eIF4E. Once 4E-BP1 is phosphorylated, EIF4G1 can effectively assemble with eIF4E and eIF4A to form the eIF4F complex, driving mRNA translation [57]. PRKAG1 is a regulatory subunit of AMPK that inhibits mTORC1 during energy stress by phosphorylating TSC2 and Raptor [58]. LARP1 is a downstream effector of mTORC1 that regulates stability and translation of mRNA [59]. USP9X has been shown to stabilize several proteins in the mTOR pathway, like mTOR, Rictor, and Raptor [60,61,62].

**Figure 3:**
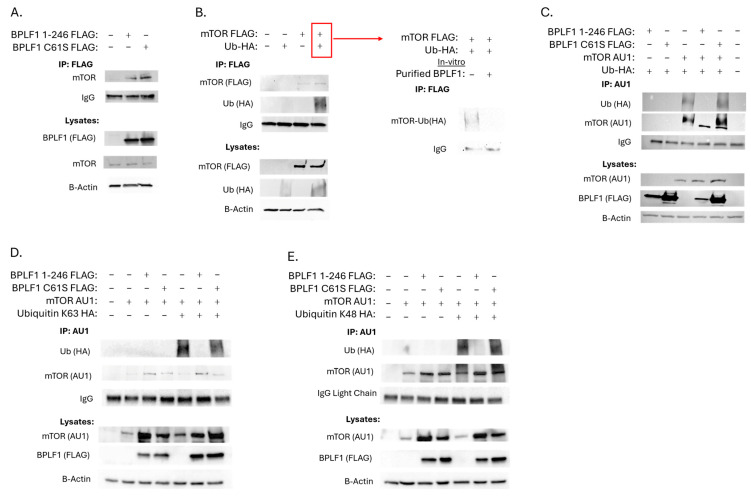
BPLF1 interacts with and deubiquitinates mTOR. (**A**). BPLF1 and mTOR interact. BPLF1 1–246 or BPLF1 1–246 C61S was overexpressed in 293T cells. Immunoprecipitation (IP) was performed using a FLAG antibody and probed for mTOR. (**B**). BPLF1 has DUB activity on mTOR in vitro. FLAG-mTOR and HA-ubiquitin were overexpressed in 293T cells. FLAG IP showed mTOR was ubiquitinated in the presence of overexpressed ubiquitin (lane 4, red box). This sample was split, and either purified BPLF1 protein or control buffer was added. After a 1-h reaction, samples were subjected to western blotting (right panel). (**C**). Total mTOR ubiquitination. AU1-mTOR, HA-ubiquitin, and either BPLF1 1–246 or BPLF1 1–246 C61S were overexpressed in 293T cells. AU1 IP showed BPLF1 1–246, but not BPLF1 1–246 C61S, reduced mTOR ubiquitination. (**D**,**E**). BPLF1 exhibits DUB activity on K63- and K48-linked ubiquitin. AU1-mTOR was expressed along with constructs that form only K63-linked or K48-linked ubiquitin chains (HA-ubiquitin K63 or K48), and BPLF1 1–246 or C61S in 293T cells. AU1 IP showed BPLF1 1–246, but not C61S, removed both K63- and K48-linked polyubiquitin from mTOR.

Initially, mTOR itself was the protein of focus, and the mass spectrometry findings of the interaction between mTOR and BPLF1 were validated by overexpressing BPLF1 1-246 or BPLF1 1-246 C61S (an enzymatically inactive construct previously described) [14,15] in 293T cells, followed by immunoprecipitation with FLAG antibody, and probing for the presence of mTOR. mTOR was found to interact with both the functional BPLF1 1-246 and non-functional BPLF1 C61S mutant (Figure 3A, lanes 2 and 3).

### 3.2. BPLF1 Deubiquitinates mTOR

To further characterize this interaction, we endeavored to determine the ubiquitination state of mTOR and whether BPLF1 could deubiquitinate mTOR in vitro. To accomplish this, mTOR, along with an HA-tagged ubiquitin construct, were overexpressed in 293T cells, and an IP for mTOR was performed. mTOR was found to be ubiquitinated in the presence of overexpressed ubiquitin, and this ubiquitination was likely polyubiquitination, as suggested by a characteristic smear (Figure 3B, lane 4). The sample shown in lane 4 (with red box) was split in two and purified BPLF1 protein was added to one and control buffer to the other. The ubiquitin seen in the control is removed from mTOR by BPLF1 (Figure 3B, right panels). This demonstrates the direct deubiquitination of mTOR by BPLF1 in vitro.

The next step was to evaluate DUB activity on mTOR in cell culture. To accomplish this, mTOR, HA-tagged ubiquitin, and either BPLF1 1-246 or BPLF1 1-246 C61S were overexpressed in 293T cells. IPs for mTOR were performed, and the results showed that BPLF1 1-246, but not the catalytically inactive BPLF1 1-246 C61S, had DUB activity on mTOR (Figure 3C, lanes 3–5). Hence, we have demonstrated BPLF1 deubiquitination of mTOR in cells and in vitro.

Linares et al. showed that mTORC1 localizes to and is activated at the lysosome only when mTOR is K63 ubiquitin tagged [36]. Activated mTORC1 then goes on to phosphorylate S6K1 and 4E-BP1 and initiate downstream processes in regulating cell growth, proliferation, and survival. To determine whether BPLF1 has DUB activity on K63-polyubiquitinated mTOR, mTOR and a ubiquitin construct that could only form K-63-linked ubiquitin chains (Ubiquitin K63 HA) were overexpressed, along with BPLF1 1-246 or BPLF1 1-246 C61S in 293T cells. IPs for AU1 tagged mTOR were performed and showed that BPLF1 1-246 but not BPLF1 1-246 C61S removed K63-linked polyubiquitin from mTOR (Figure 3D).

The same procedure was used to assess whether BPLF1 could remove K48-ubiquitin linkages from mTOR, which typically signals mTOR to be degraded at the proteasome. mTOR and a ubiquitin construct that could only form K48- linked ubiquitin chains (Ubiquitin K48 HA) were overexpressed, along with BPLF1 1-246 or BPLF1 1-246 C61S in 293T cells. IPs for AU1-tagged mTOR were performed, and it was found that BPLF1 1-246 but not BPLF1 1-246 C61S had DUB activity on K-48-linked polyubiquitinated mTOR (Figure 3E). Taken together, these results suggest that BPLF1 reduces mTORC1 activation via K63-linked deubiquitination and that BPLF1 can rescue mTOR from proteasomal degradation.

### 3.3. BPLF1 Decreases Activation of mTORC1

**Figure 4:**
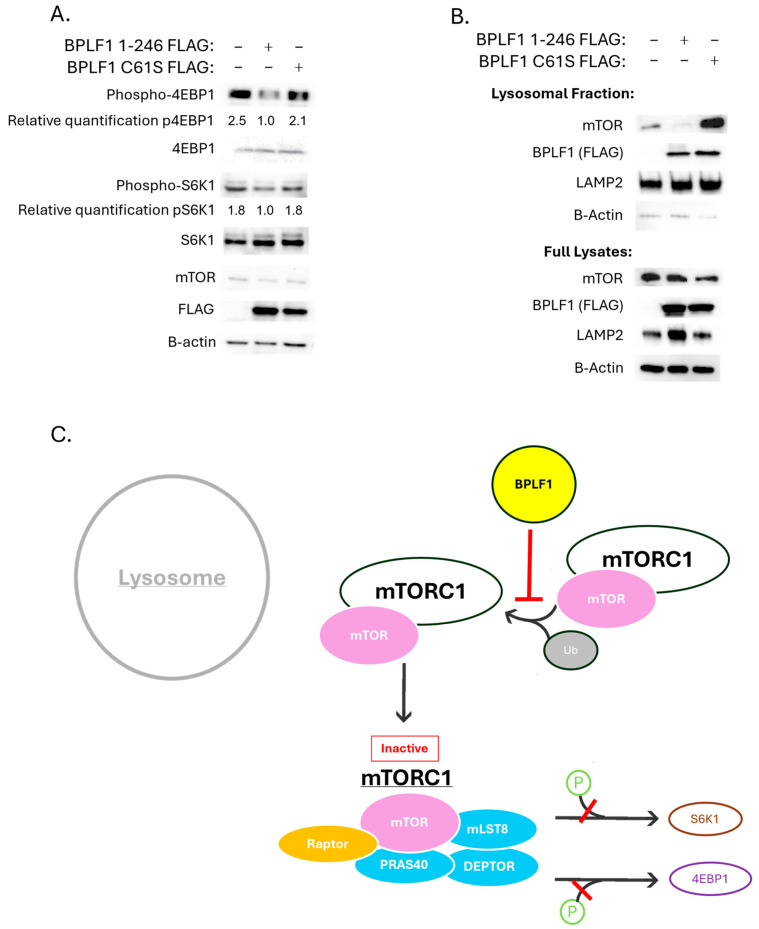
BPLF1 decreases mTORC1 activation. (**A**). BPLF1 reduces phosphorylation of mTORC1 downstream effectors. BPLF1 1–246 or BPLF1 1–246 C61S was overexpressed in 293T cells. Western blotting was performed to detect phosphorylated forms of S6K1 and 4EBP1 and compared to total protein levels. Average relative expression levels of phospho-4EBP1 and phospho-S6K1 are listed below their corresponding bands. (**B**). BPLF1 decreases mTOR localization to the lysosome. BPLF1 1–246 or BPLF1 1–246 C61S was overexpressed in 293T cells. Lysosomal fractions were isolated, and western blotting was performed to detect mTOR and the lysosomal marker LAMP2. (**C**). Proposed mechanism for BPLF1’s effect on mTORC1 activation.

BPLF1’s DUB activity on K63-linked polyubiquitination of mTOR led to the investigation of whether there was a change in mTORC1 activation in the presence of BPLF1. To assess the activation state of mTORC1, the phosphorylation levels of downstream targets of mTORC1, S6K1 and 4E-BP1, were evaluated and compared to the total levels of each protein. The presence of functional BPLF1 1-246 reduced the phosphorylation levels of 4E-BP1 and, to a lesser extent, S6K1. The inactive BPLF1 C61S mutant did not result in a discernible decrease in the phosphorylation of S6K1 and 4E-BP1, indicating that BPLF1 DUB activity results in decreased mTORC1 downstream activity (Figure 4A).

Given that BPLF1 1-246 causes a decrease in mTORC1 downstream signaling, the next step was to determine whether BPLF1’s removal of K63-linked ubiquitin from mTOR reduces mTORC1 localization to the lysosome, the site of its activation. To evaluate this, lysosomal fractionation was performed after BPLF1 1-246 or BPLF1 1-246 C61S was overexpressed in 293T cells. Figure 4B shows that mTOR localization to the lysosome was reduced in the presence of BPLF1 1-246, but not with the C61S mutant (lane 2 vs. lane 3). These data demonstrate that K63-linked deubiquitination of mTOR by BPLF1 disrupts mTORC1 localization to the lysosome, thereby inhibiting its activation and subsequent phosphorylation of S6K1 and 4E-BP1.

Taken together, these data show that BPLF1’s DUB activity on mTOR, particularly its removal of K63-linked ubiquitin chains, prevents mTORC1 localization and activation at the lysosome, reducing the phosphorylation of downstream effectors, and ultimately dysregulating cellular processes associated with growth, proliferation, and survival (Figure 4C).

### 3.4. BPLF1 Interacts with Raptor

Raptor, another component of the mTOR pathway, was identified as a significant BPLF1 interactor in the mass spectrometry screen (Table 1). Hussain et al. previously reported that the endogenous DUB UCH-L1 disrupts the interaction between Raptor and the DDB1-CUL4 ubiquitin ligase complex, preventing DDB1-CUL4-mediated Raptor ubiquitination. This cascade of events, which leaves raptor not ubiquitinated, promotes the dissolution of mTORC1 and increased formation of mTORC2 [63].

**Figure 5:**
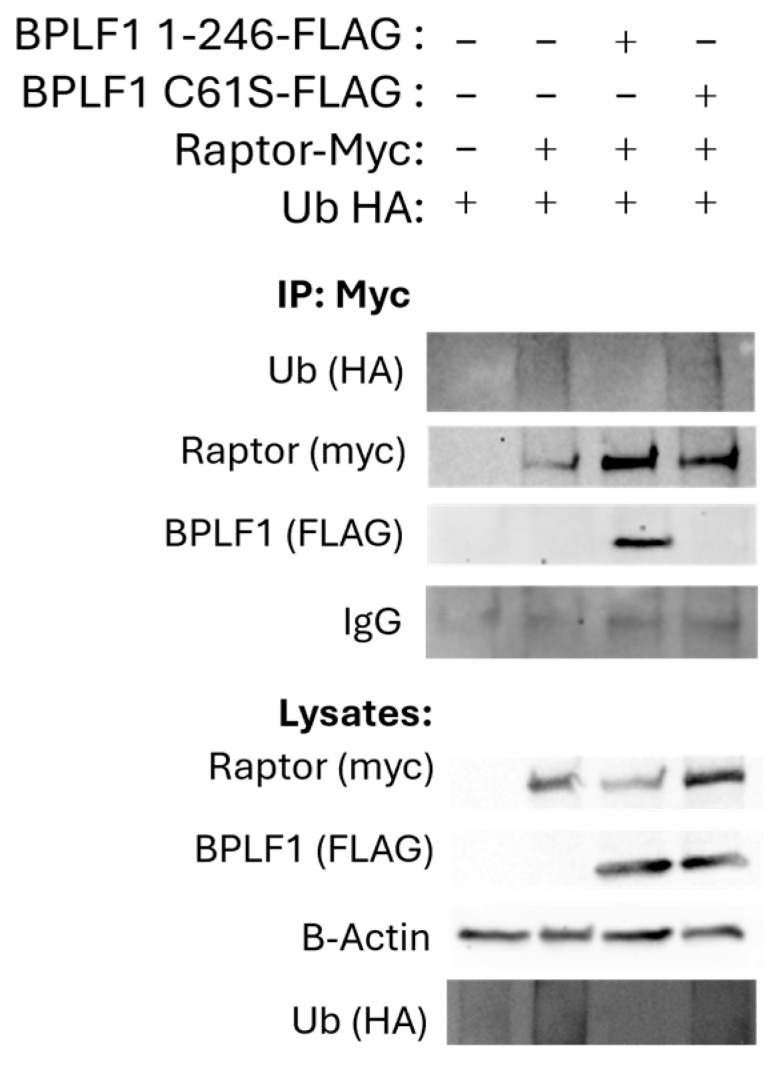
BPLF1 deubiquitinates Raptor. Myc-Raptor and HA-ubiquitin were co-expressed with BPLF1 1–246 or C61S in 293T cells. An IP for Myc showed BPLF1 1–246, but not the C61S mutant, had DUB activity on Raptor.

To assess whether BPLF1 has DUB activity on Raptor, myc-Raptor and HA-tagged ubiquitin were co-expressed with either BPLF1 1-246 or the BPLF1 1-246 C61S mutant in HEK293T cells. An IP for Raptor confirmed its interaction with BPLF1 and showed that BPLF1 1-246, but not the C61S mutant, deubiquitinates Raptor (Figure 5, lanes 3 and 4).

Since BPLF1 expression decreases the ubiquitination of both Raptor and mTOR, the question arose whether, as seen with UCH-L1, this would result in reduced mTORC1 and a corresponding shift toward mTORC2 [36,63,64]. Therefore, the hypothesis was that BPLF1’s DUB activity was not only preventing mTORC1 lysosomal localization and activation, but also causing the dissolution of mTORC1 and in turn causing a shift toward the increased formation of mTORC2.

### 3.5. BPLF1 Disrupts mTORC1 but Not mTORC2 Complex Formation

**Figure 6:**
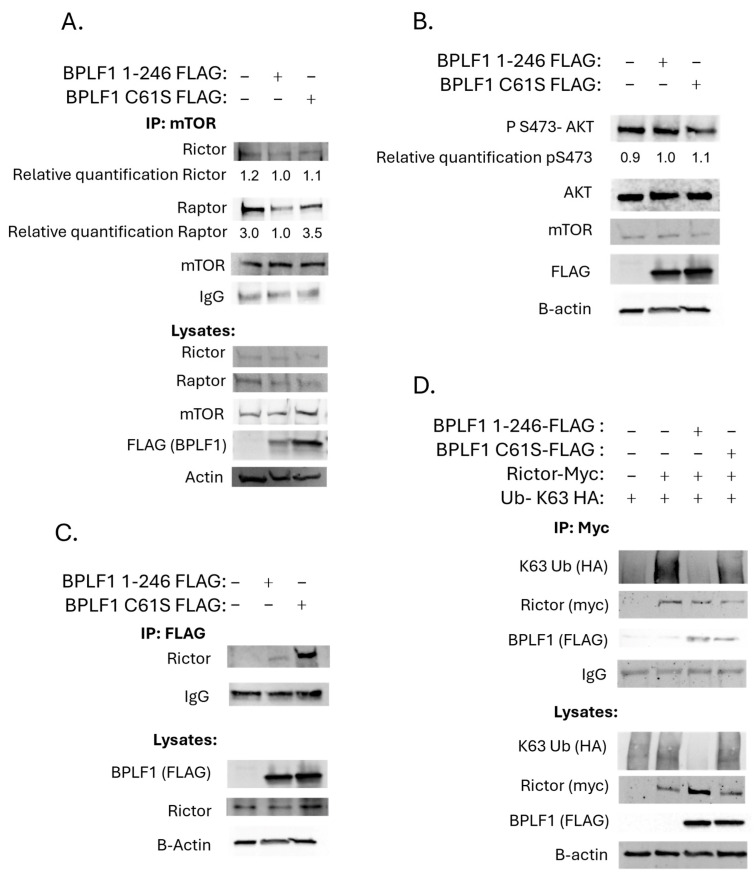
BPLF1 effects on mTOR complex formation. (**A**). BPLF1 disrupts mTORC1 but not mTORC2 complex formation. BPLF1 1–246 or BPLF1 1–246 C61S was overexpressed in HeLa cells. mTOR was immunoprecipitated, and co-IP for Raptor (mTORC1) and Rictor (mTORC2) was assessed by western blot. Average relative expression levels of Raptor and Rictor are listed below their corresponding bands. (**B**). BPLF1 did not reduce mTORC2 downstream signaling. BPLF1 1–246 or C61S was overexpressed in HEK293T cells. Western blotting was performed to detect phosphorylated AKT (S473) levels compared to total AKT. Average relative expression levels of pS473-AKT are listed below the corresponding bands. (**C**). BPLF1 interacts with Rictor. BPLF1 constructs were overexpressed in 293T cells. FLAG IP was performed and probed for Rictor. (**D**). BPLF1 has DUB activity on Rictor. Myc-Rictor, HA-Ubiquitin K63, and either BPLF1 1–246 or C61S mutant were overexpressed in HEK293T cells. Myc IP showed that BPLF1 1–246, but not C61S, removed K63-linked polyubiquitin from Rictor.

To assess whether BPLF1 expression shifts the balance from mTORC1 to mTORC2, BPLF1 1-246 or the catalytically inactive BPLF1 1-246 C61S mutant were overexpressed in HeLa cells, followed by immunoprecipitation (IP) for mTOR. Raptor co-immunoprecipitation was used as a marker for mTORC1, and Rictor co-immunoprecipitation as a marker for mTORC2, since Raptor is exclusive to mTORC1 and Rictor to mTORC2. Western blot analysis revealed that BPLF1 1-246, but not the C61S mutant, reduced Raptor levels (Figure 6A, lane 2), indicating a decrease in mTORC1 complex formation. Interestingly, mTORC2 levels, as indicated by Rictor expression, remained largely unchanged in the presence of either BPLF1 construct. A singular experiment was also performed in 293T cells and a similar trend was observed (Supplementary Figure S2).

To further explore mTORC2 activity, the phosphorylation of AKT at S473 was evaluated, as it is a known downstream target of the active mTORC2 kinase. Neither BPLF1 1-246 nor BPLF1 1-246 C61S changed S473-AKT phosphorylation (Figure 6B). These results, in our hands, did not show an increase in mTORC2 activation, as monitored through AKT phosphorylation, even though mTORC1 complex formation was downregulated. Not observing a change in mTORC2 in the presence of BPLF1 was a surprising result, considering the literature on Raptor deubiquitination described above [63].

Rictor’s ubiquitination status is also known to regulate mTORC2 assembly. Wrobel et al. demonstrated that the endogenous DUB USP9X removes K63-linked ubiquitin from Rictor, enhancing its interaction with mTOR. Loss of USP9X led to decreased mTORC2 activity due to the impaired ability of Rictor and mTOR to associate [60]. Although Rictor did not appear in the mass spectrometry screen, an IP confirmed an interaction between BPLF1 and Rictor (Figure 6C). To determine whether BPLF1 could remove K63-linked ubiquitin from Rictor, a K63-only ubiquitin construct was overexpressed with Rictor and either BPLF1 1-246 or the C61S mutant in 293T cells. IPs for Rictor showed that BPLF1 1-246, but not BPLF1 1-246 C61S, removed K63-linked polyubiquitin from Rictor (Figure 6D).

### 3.6. Inhibition of mTORC1 Increases Infectious Virus Production of EBV

Having established that BPLF1 downregulates mTORC1 activation and downstream signaling, we questioned whether inhibition of mTORC1 would affect EBV infectious virus production. Previous studies have established various roles of mTORC1 in different EBV-mediated diseases such as NPC, B-cell lymphomas, and gastric carcinoma [44,47,48,65]. To assess the importance of mTORC1 in infectious virus production, a GFP-293T EBV+ cell line was used as previously described [51]. Cells were treated with rapamycin (0.5 nM and 2 nM), an mTORC1 inhibitor, during lytic reactivation induced by BZLF1 transfection. After 48 h, supernatants containing GFP-tagged viral particles were collected, cleared of debris, and concentrated to equivalent volumes. Equal volumes (100 µL) of supernatant fluids were used to infect Raji cells, and EBV infectivity was assessed 48 h post-infection via flow cytometry for GFP. Surprisingly, rapamycin treatment increased infectious EBV production by 5–6-fold.

**Figure 7:**
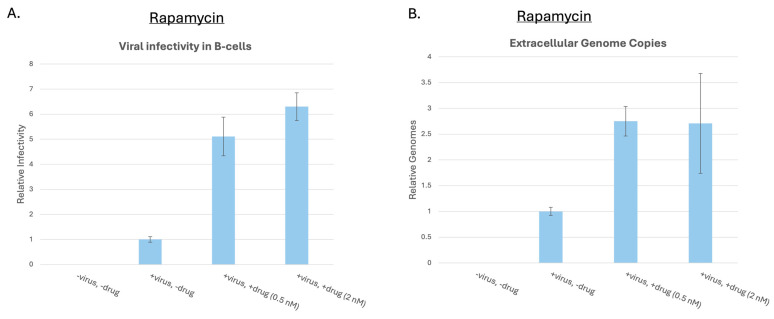
Inhibition of mTORC1 increases EBV infectious virus production. (**A**). Inhibition of mTORC1 with rapamycin. 293EBV+ cells were treated with rapamycin (0.5 nM and 2 nM) during induction of the lytic cycle. After 48 h, GFP+ virions in the supernatant were used to infect Raji cells. Virus production was determined by GFP detection and normalized to the untreated control. Error bars represent SEM. (**B**). qPCR quantification of extracellular EBV genomes. Supernatants were treated with DNase to remove unencapsulated DNA. Encapsulated viral DNA was extracted and quantified using qPCR. Error bars represent SEM.

To confirm this effect, viral supernatants were treated with DNase to eliminate unencapsulated DNA (as enveloped EBV virions are DNase-resistant), followed by DNA extraction and qPCR to quantify extracellular viral genome copies (Figure 7B). The increase in EBV genome copy number upon rapamycin treatment mirrored the infectivity data (Figure 7A). These findings suggest that BPLF1-mediated inhibition of mTORC1 promotes EBV infectivity.

### 3.7. mTORC2 Inhibition with JR-AB2-011 Decreases EBV Infectious Virus Production

**Figure 8:**
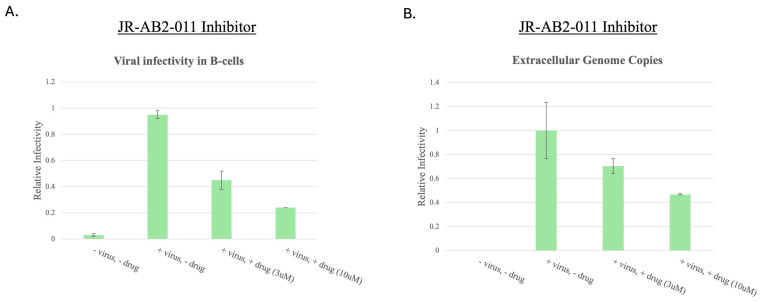
Inhibition of mTORC2 decreases EBV infectious virus production. (**A**). Inhibition of mTORC2 with JR-AB2-011. 293EBV+ cells were treated with JR-AB2-011 (3 µM and 10 µM) during induction of the lytic cycle. After 48 h, GFP+ virions in the supernatant were used to infect Raji cells. Virus production was determined and normalized as in Figure 7. Error bars represent SEM. (**B**). qPCR quantification of extracellular EBV genomes. Supernatants were treated with DNase, and encapsulated viral DNA was extracted and quantified. Error bars represent SEM.

Despite previous results indicating that BPLF1 did not impact mTORC2 formation or signaling, we hypothesized that mTORC2 still plays a critical role in EBV infection. To test this, we treated GFP-293T EBV+ cells with JR-AB2-011 (3 µM and 10 µM), a selective mTORC2 inhibitor. Following the same protocol, virus-containing supernatants were used to infect Raji cells, and infectivity was quantified by flow cytometry. Inhibition of mTORC2 led to a >70% reduction in EBV infectivity at 10 µM JR-AB2-011 (Figure 8A).

To confirm this result, DNase-treated supernatants were analyzed using qPCR to measure the number of extracellular EBV genome copies. A corresponding decrease in viral genome production was observed with JR-AB2-011 treatment (Figure 8B). Although BPLF1 alone did not alter mTORC2 activity, these results suggest that mTORC2 is required for optimal EBV production. It is possible that other viral factors or alternative mechanisms cooperate with BPLF1 to regulate mTORC2 during infection.

## 4. Discussion

BPLF1 has been established as critical to many of EBV’s processes including DNA repair, immune evasion, viral infectivity, B-cell immortalization, and tumor formation [14,15,16,17,18,19,20,21,22]. BPLF1 has been shown to interact with numerous cellular and viral proteins [66]. This study expands on previous work and identifies BPLF1 as an important regulator of mTORC1 signaling through its DUB activity on mTOR, a possible or partial mechanism for how BPLF1 enables EBV infectivity and viral replication.

**Figure 9:**
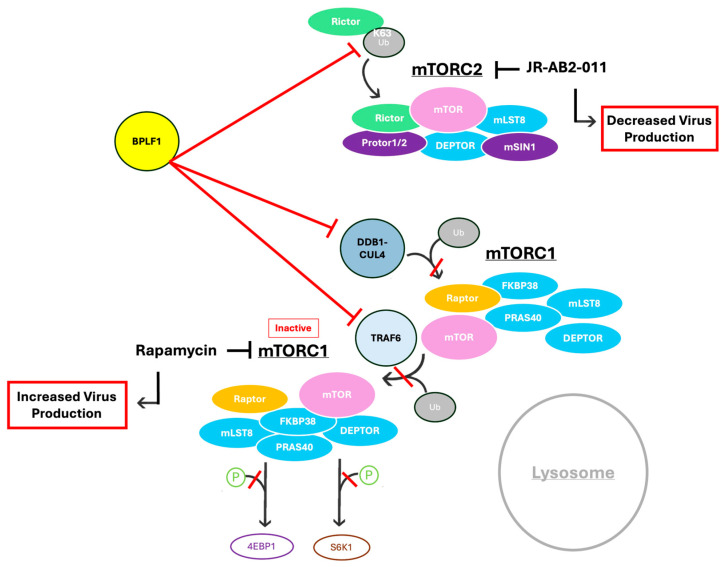
Proposed mechanism of BPLF1’s effect on the mTOR pathway. BPLF1 interacts with and deubiquitinates mTOR, Raptor, and Rictor. It decreases mTORC1 localization and activation at the lysosome and suppresses mTORC1 downstream signaling via S6K1 and 4EBP1. Inhibition of mTORC1 increased infectious virus production, whereas inhibition of mTORC2 decreased it.

The affinity-based mass spectrometry screen led to the understanding that BPLF1 interacts with and deubiquitinates mTOR and various proteins linked to mTOR signaling, including Raptor and Rictor. Further, we found that BPLF1 acts on mTORC1 by decreasing its localization and activation at the lysosome and downstream signaling through S6K1 and 4E-BP1 (Figure 9). These data are supported by established knowledge that mTOR is extensively regulated by ubiquitin post-translational modifications, including TRAF6 signaling mTORC1 activation at the lysosome through its E3 ligase activity [36]. Interestingly, BPLF1 has been shown to interact with and inhibit TRAF6 activation, another mechanism by which BPLF1 may be preventing mTORC1 activation [17].

Our conclusions on the effect of BPLF1 on the mTOR pathway are further bolstered by the observed DUB activity of BPLF1 on Raptor, a key mTORC1 complex component. By deubiquitinating Raptor, BPLF1 could be contributing to mTORC1 downregulation through an additional mechanism not related to the mTOR K63-mediated ubiquitination necessary for lysosomal localization. Our findings showing Raptor deubiquitination align with prior studies that link Raptor deubiquitination to reduced mTORC1 signaling [63,67]. Further, it has been shown that BPLF1 has deneddylase activity on CUL4, which when deneddylated, disrupts the E3 ligase complex it forms [68]. Since Raptor is ubiquitinated by the DDB1-CUL4 complex to form mTORC1, this deneddylase activity provides another avenue by which BPLF1 may be disrupting mTORC1 formation [68,69]. In previous literature, there has been evidence that DUB activity on Raptor, and mTORC1 downregulation as a whole, might cause a secondary shift toward mTORC2 [63,67].

Additionally, we found that BPLF1 can deubiquitinate Rictor, the mTORC2 protein which has been shown to only associate with mTOR to form mTORC2 when K63 ubiquitin is removed by the endogenous DUB USPX9 [60]. USP9X expression seems to promote a shift from mTORC1 to mTORC2 formation, indicating there may be a “zero sum game” between mTOR complexes. This was further exemplified in a study when knockdown of USP9X amplified mTORC1 activity [61]. This confirmed the expectation that, in addition to the downregulation of mTORC1, BPLF1 would cause a secondary upregulation of mTORC2. Despite this hypothesis, surprisingly, under our conditions, no noticeable changes in mTORC2 signaling were observed. This suggests that BPLF1 may be disrupting mTORC1 without directly enhancing mTORC2 signaling.

Despite the contradictory nature of our observation, that BPLF1 did not promote mTORC2 even though Rictor deubiquitination was observed, compared to previously published literature, it is important to keep in mind that there are many known and possibly even additional unelucidated proteins which interact with mTOR and BPLF1 and contribute to their regulation. One example of another possible confounding factor is the E3 ligase FBX8 which has been shown to target mTOR for ubiquitin-mediated degradation, a process shown to be essential for FBX8-induced cell proliferation and invasion in colorectal cancer (CRC) [70]. Additionally, the previously discussed cell-dependent effect of rapamycin on the lytic cycle allows for the further question of whether there are other cell-dependent considerations when studying the mTOR pathway in EBV [48]. Further studies should consider expanding to include experiments in B-cells to assess whether there are differential changes of BPLF1 on the mTOR pathway.

Further, Raptor appeared as an interacting partner in the mass spectrometry screen when BPLF1 was expressed alone without viral reactivation; however, during viral reactivation, this interaction was not detected. Furthermore, unlike Rictor and mTOR, where BPLF1 WT and C61S interacted with both proteins, only BPLF1 WT, and not BPLF1 C61S, interacted with Raptor. Interestingly, Rictor did not appear on the mass spectrometry screen at all as a significant interacting partner, but we did see the interaction in our studies (Figure 6C). Observed interactions seen in our experiments may be indirect or have another confounding factor. It would be interesting to further elucidate both the BPLF1-Raptor and the BPLF1-Rictor interactions.

Both mTORC1 and mTORC2 inhibitors have been used to treat various cancers, with mTORC1 inhibitors having limited therapeutic efficacy and mTORC2 inhibitors having greater efficacy [47]. mTORC1 and mTORC2 have extensive feedback mechanisms and interrelated regulation, so each pathway’s role in cancer is very difficult to parse out [47].

We observed in our experiments that inhibiting mTORC1 with rapamycin increased EBV infectious virus production. This result demonstrates the importance of mTORC1 regulation in EBV’s life cycle. Taken together with our other data, this suggests that BPLF1-mediated downregulation of mTORC1 may create a cellular environment that is better suited for viral replication and release.

Conversely, inhibition of mTORC2 with JR-AB2-011, a drug that disrupts the association of mTOR and Rictor in the formation of mTORC2, reduced EBV infectious virus production. This demonstrates a distinct and necessary role for mTORC2 in EBV replication independent of our observed findings that suggest BPLF1 activity does not promote mTORC2 formation or affect downstream signaling. However, the decreased infectious virus production observed with JR-AB2-011 treatment is interesting considering previously published work that observed that the inhibition of the downstream Akt pathway increased the effects of rapamycin in treating EBV+ B-cell lymphomas [50]. The interplay between these mTOR pathways may be important to better understanding how to treat EBV+ cancers.

Taken together, both in our work and previously published work, it is clear that mTOR’s role in EBV infectivity is substantial [49]. It is particularly striking how EBV can selectively manipulate the regulation of one single protein, mTOR, by using post-translation modifications to selectively downregulate one of mTOR’s functions while upregulating others using effector viral proteins, such as BPLF1. The complexity of the way the virus co-opts cellular processes and functions highlights the need to better understand the mechanisms by which the virus enacts its pathogenicity in order to effectively treat and eventually cure EBV-associated disease.

To conclude, BPLF1’s disruption of mTORC1 likely enhances EBV infectivity by promoting an intracellular environment that favors viral progeny release. While the critical role of mTORC1 inhibition is clear, the relationship between BPLF1 and mTORC2 requires further investigation of other potential viral factors that may contribute to mTORC2 related viral processes. As mentioned above, several other mTOR pathway interacting partners from the mass spectrometry analysis may also serve important roles in mTOR regulated processes and investigating the role of BPLF1 on each one would likely further elucidate the mechanism by which BPLF1 is aiding EBV infectivity through mTOR mediated mechanisms. Understanding the molecular mechanisms by which BPLF1 alters mTOR signaling may offer novel insights into EBV pathogenesis and potential therapeutic targets for EBV-associated diseases.

## Figures and Tables

**Table 1:** Mass Spectrometry data interacting partners of BPLF1 related to the mTOR Pathway. 293EBV+ cells were transfected with BPLF1 full-length (FL) or BPLF 1-246 FLAG-tagged expression constructs with and without induction of the virus lytic cycle, as indicated. Scoring was normalized to control samples where BPLF1 1-246 and full-length BPLF1 were not expressed. STRING analysis showed several BPLF1 interacting partners known to be associated with mTOR signaling. mTOR, Raptor, TelO2, TTI1, SMG1, TSC2, EIF4G1, PRKAG1, LARP1 and USP9X. Log2(FC) sample vs. Control (with no BPLF1 expressed) value was determined for significance; a value above 1.0 was deemed significant.

Protein (Interacting with BPLF1)	Protein Function in mTOR Pathway	No Virus Induction with FLAG-BPLF1 FL Expression (Log2(FC))	No Virus Induction with FLAG-BPLF1_1-246 Expression (Log2(FC))	Virus Induction with FLAG-BPLF1 FL Expression (Log2(FC))	Virus Induction with FLAG-BPLF1_1-246 Expression (Log2(FC))
mTOR	Protein kinase responsible for controlling cellular metabolism, catabolism, immune responses, autophagy, survival, proliferation, and migration, to maintain cellular homeostasis.	2.17	4.56	2.33	4.69
Raptor	Regulates mTORC1 formation and signaling.	1.52	3.03	NA	NA
TELO2	Stabilize mTOR complexes and regulate mTOR signaling.	1.60	4.33	2.28	4.63
TELO2-IP 1	Stabilize mTOR complexes and regulate mTOR signaling.	1.22	5.60	1.90	4.03
SMG1	Functions related to the mTOR signaling pathway	−0.89	3.13	NA	3.36
TSC2	Regulation of the common mTOR pathway of protein synthesis, cell growth, and viability in response to cellular energy levels	2.97	5.50	3.43	5.24
EIF4G1	Has mTOR signaling mediated tumorigenesis-promoting functions.	0.93	3.65	1.41	3.35
PRKAG1	Subunit of the AMPK protein kinase, which is involved in regulating cell growth and energy metabolism.	0.74	2.46	−0.05	1.69
LARP1	RNA-binding protein that functions as a molecular switch for mTORC1-mediated translation of mRNAs.	0.12	−0.36	1.19	0.15
USP9X	Negatively regulates mTOR activity.	0.98	3.52	1.35	3.50
BPLF1	EBV encoded DUB enzyme	13.32	8.76	12.83	7.77

## Data Availability

All data and Supplementary Information are included in this published work.

## Supplementary Materials

The following supporting information can be downloaded at: https://www.mdpi.com/article/10.3390/v17081139/s1, Figure S1: Functional activity of BPLF constructs; Figure S2: BPLF1 effects on mTOR complex formation; Table S1: BPLF1 Mass Spectrometry Detection of Interacting Proteins.
