# Supplementary figures and images for: Novel Role of the Epstein-Barr Virus Encoded Deubiquitinating Enzyme (BPLF1) in mTOR-Mediated Cell Growth and Proliferation Pathways
Source: Viruses. 2025 Aug 20;17(8):1139. doi: 10.3390/v17081139 (PMC12390678; doi:10.3390/v17081139)

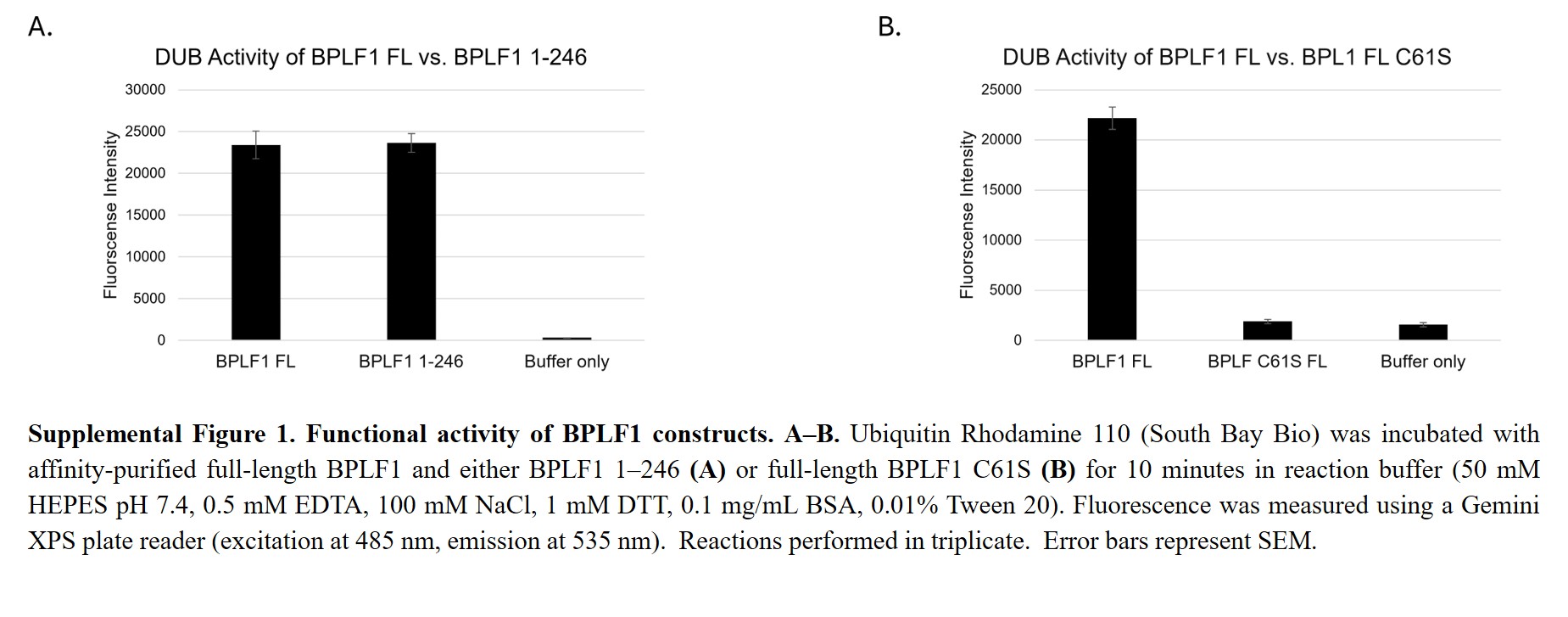

Supplement: Supplementary file 1 [file viruses-17-01139-s001.zip › supplementary1mund.jpg]

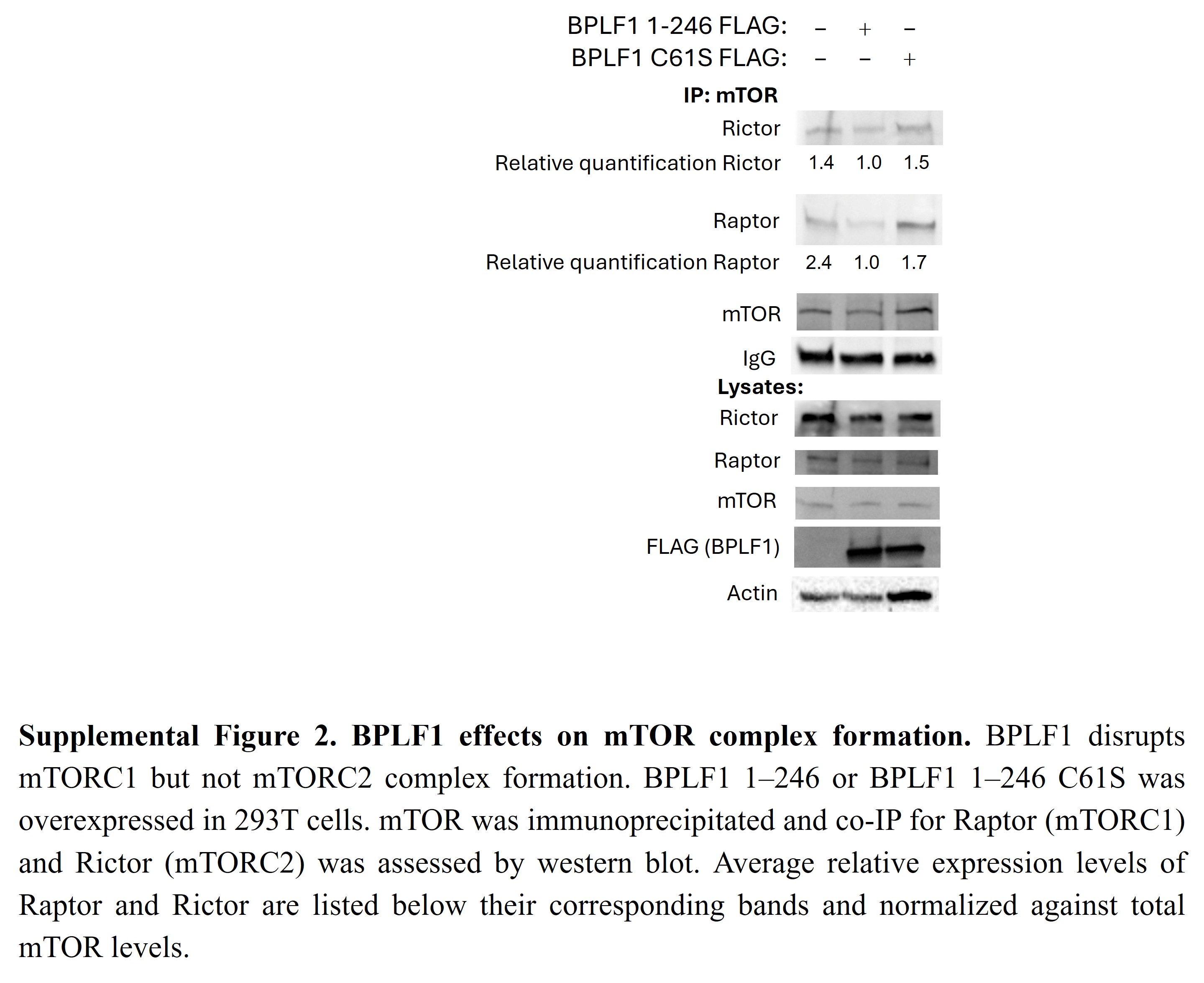

Supplement: Supplementary file 1 [file viruses-17-01139-s001.zip › supplemental2mund.jpg]
